# A novel risk classifier to predict the in-hospital death risk of nosocomial infections in elderly cancer patients

**DOI:** 10.3389/fcimb.2023.1179958

**Published:** 2023-05-10

**Authors:** Aimin Jiang, Yimeng Li, Ni Zhao, Xiao Shang, Na Liu, Jingjing Wang, Huan Gao, Xiao Fu, Zhiping Ruan, Xuan Liang, Tao Tian, Yu Yao

**Affiliations:** Department of Medical Oncology, The First Affiliated Hospital of Xi’an Jiaotong University, Xi’an, Shaanxi, China

**Keywords:** cancer patients, nosocomial infections, prognostic nutritional index, nomogram, mortality

## Abstract

**Background:**

Elderly cancer patients are more predisposed to developing nosocomial infections during anti-neoplastic treatment, and are associated with a bleaker prognosis. This study aimed to develop a novel risk classifier to predict the in-hospital death risk of nosocomial infections in this population.

**Methods:**

Retrospective clinical data were collected from a National Cancer Regional Center in Northwest China. The Least Absolute Shrinkage and Selection Operator (LASSO) algorithm was utilized to filter the optimal variables for model development and avoid model overfitting. Logistic regression analysis was performed to identify the independent predictors of the in-hospital death risk. A nomogram was then developed to predict the in-hospital death risk of each participant. The performance of the nomogram was evaluated using receiver operating characteristics (ROC) curve, calibration curve, and decision curve analysis (DCA).

**Results:**

A total of 569 elderly cancer patients were included in this study, and the estimated in-hospital mortality rate was 13.9%. The results of multivariate logistic regression analysis showed that ECOG-PS (odds ratio [OR]: 4.41, 95% confidence interval [CI]: 1.95-9.99), surgery type (OR: 0.18, 95%CI: 0.04-0.85), septic shock (OR: 5.92, 95%CI: 2.43-14.44), length of antibiotics treatment (OR: 0.21, 95%CI: 0.09-0.50), and prognostic nutritional index (PNI) (OR: 0.14, 95%CI: 0.06-0.33) were independent predictors of the in-hospital death risk of nosocomial infections in elderly cancer patients. A nomogram was then constructed to achieve personalized in-hospital death risk prediction. ROC curves yield excellent discrimination ability in the training (area under the curve [AUC]=0.882) and validation (AUC=0.825) cohorts. Additionally, the nomogram showed good calibration ability and net clinical benefit in both cohorts.

**Conclusion:**

Nosocomial infections are a common and potentially fatal complication in elderly cancer patients. Clinical characteristics and infection types can vary among different age groups. The risk classifier developed in this study could accurately predict the in-hospital death risk for these patients, providing an important tool for personalized risk assessment and clinical decision-making.

## Introduction

Nowadays, with the dramatic development of the economy and lifestyle changes, cancer has become a major public health problem that threatens human life all over the world (Siegel et al., 2023). According to the latest cancer statistics, there will be an estimated two million newly diagnosed cases of cancer in the United States in 2023, with an estimated 609,820 cancer-related deaths also expected (Siegel et al., 2023). Cancer has become a significant public health challenge in China since 2010, overtaking all other causes of death in the country (Chen et al., 2016). Even though significant advances in cancer diagnosis and treatment have been made in recent decades, the incidence of anti-tumor-related adverse events is gradually increasing. Among these adverse events, nosocomial infections are common and can result in higher mortality rates in cancer patients (Baden et al., 2016). Patients with malignancies are more vulnerable to developing severe infections due to immunosuppression caused by surgery, radiotherapy, and long-term cytotoxic treatment (Maschmeyer and Haas, 2008; Brand et al., 2016; Gudiol et al., 2016; Taplitz et al., 2018). Besides, more frequent exposure to various invasive procedures also significantly augmented this risk. Consequently, nosocomial infections not only disrupted the anticipated cancer treatment schedule but increased their healthcare-related economic burden and risk of death (Kamboj and Sepkowitz, 2009; Brand et al., 2016).

As global aging intensifies, the number and proportion of the elderly population are increasing rapidly in almost every country in the world. Elderly cancer patients are an especially vulnerable population with an extremely high risk of malnutrition and developing nosocomial infections during anti-cancer treatment (Aydemir et al., 2013; Brand et al., 2016; Antonio et al., 2019; Li et al., 2023). Previous publications have revealed that the mortality of bacteremia in elderly cancer patients was as high as ~18% (Aydemir et al., 2013; Antonio et al., 2019). Besides, it is reported that elderly cancer patients have a higher 30-day mortality than younger patients after bloodstream infection (BSI) (Antonio et al., 2019). Although numerous studies have well described the microbiological characteristics and prognostic factors of nosocomial infections in cancer patients, only limited studies have focused on elderly cancer patients (Aydemir et al., 2013; Antonio et al., 2019; Li et al., 2023). Most importantly, to date, no risk model has been developed to predict the prognosis of nosocomial infections in elderly cancer patients. In our previous work, we systematically explored the clinical characteristics, microbiological distribution, and prognostic factors of nosocomial infections in cancer patients through a large-scale retrospective study (Jiang et al., 2020b; Jiang et al., 2022). Here we constructed a novel and reliable risk classifier that could effectively predict the in-hospital death risk of nosocomial infections in elderly cancer patients.

## Methods

### Study design and data source

All data used in this study were obtained from the Xi’an Jiaotong University Cancer and Infection Cohort (XJUCIC), which is a large-scale single-center retrospective cohort study conducted from August 2013 to May 2019 with the aim of exploring the clinical characteristics, microbiological distribution, and risk factors of nosocomial infections in cancer patients (Jiang et al., 2022). The primary objective of the current study was to investigate the characteristics of nosocomial infections in elderly cancer patients and develop a novel clinical predictive model to predict the in-hospital death risk of nosocomial infections in these participants. The definition and the inclusion and exclusion criteria of the participants were described in a previous study (Jiang et al., 2022). The study outcome was in-hospital mortality resulting from nosocomial infections, and did not take into account cancer-related deaths or death events caused by other factors. The ethics committee of the Frist Affiliated Hospital of Xi’an Jiaotong University approved this study (No: XJTU1AF2020LSK-049). Besides, we conducted this study under the requirement of the declaration of Helsinki.

### Data processing and cohort establishment

The demographic data collected in this study included age, gender, smoking history, Charlson comorbidity index (CCI) (Charlson et al., 1987), and common complicated diseases. Cancer-related information, such as malignancy types, Eastern Cooperative Oncology Group performance status (ECOG-PS), the 8^th^ edition of the American Joint Committee on Cancer (AJCC)-TNM staging, and antineoplastic treatment options, were also recorded. Infection-related information comprised the source of infections, presence of fever, length of antibiotics treatment, intensive care unit (ICU) admission and the experience of septic shock and ventilator intervention. The study also collected relevant laboratory examination results, including blood routine tests, serum albumin level, and serum procalcitonin (PCT), and documented the microorganisms cultured from each participant.

Elderly cancer patients were referred as cancer patients older than 60 years old (Gong et al., 2020). To compare the clinical characteristics and infection features across different age groups, the patients were further categorized into three groups: 60-69 years old, 70-79 years old, and ≥80 years old. The prognostic nutritional index (PNI) was calculated according to the following formula:


$$\text{PNI}=\text{serum albumin level }\left(\text{g}/\text{L}\right)+5*\text{peripheral lymphocyte count }\left(10^{9}/\text{L}\right)$$

The optimal cut-off value for PNI was determined using the “roc” function in the “pROC” package, and a dichotomous variable was created based on this cut-off value (36.625, **Figure S1**). The entire cohort was divided into a training cohort (341 cases) and a validation cohort (228 cases) using a 6:4 ratio, as determined by the “initial_split” function in the “rsample” package. The training cohort was used for model development, while the validation cohort was used to evaluate the model’s discrimination and calibration abilities.

### Feature selection

Given the high dimensionality of the dataset, we applied the Least Absolute Shrinkage and Selection Operator (LASSO) algorithm to filter the optimal variables for model development and avoid model overfitting (Ghosh et al., 2015). By exploiting the penalty parameter lambda, LASSO regression could shrink the coefficients of unimportant variables to zero, thus filtering the important variables. The optimal lambda value was determined by 10-fold cross-validation (CV). LASSO regression was achieved via the “glmnet” package (Engebretsen and Bohlin, 2019), and the “dummyVars” function was utilized to achieve One-Hot encoding for categorical variables. Afterward, variables with non-zero coefficients were selected for the univariate logistic regression analysis. Ultimately, variables with a *P* value<0.05 in the univariate analysis were adopted into the multivariate logistic regression analysis to identify the independent predictors of in-hospital death risk of nosocomial infections in elderly cancer patients.

### Nomogram development and evaluation

The independent predictors identified in the multivariate logistic regression analysis were used to develop a nomogram for predicting the in-hospital death risk of each elderly cancer patient who experienced nosocomial infections. The “rms” package was used for nomogram visualization, and the total points of each participant were obtained using the “nomogramFormula” package. Then, the receiver operating characteristics curve (ROC) was used to evaluate the discrimination ability of the nomogram by estimating the area under the curve (AUC). The bootstrap method was used to compare the AUC of different predictors via the “pROC” package (Robin et al). The calibration curve was also adopted to assess the calibration ability of the model, with the Hosmer and Lemeshow goodness of fit (GOF) test performed to test the consistency between the actual and the predicted in-hospital death risk (Hosmer et al., 2013). Given that the discrimination and calibration abilities of the nomogram could not reflect the “false positive” and “false negative” events in the dataset, we further conducted decision curve analysis (DCA) to evaluate the net clinical benefit of the nomogram (Kerr et al., 2016). Finally, the performance of the nomogram was verified using the validation cohort as an internal validation cohort.

### Statistical analysis

Continuous variables were described as means and standard deviation (SD) or median and interquartile (IQR) as appropriate. Categorical variables were summarized as count and percentage and were analyzed by the chi-square or Fisher’s exact tests as appropriate. Two independent sample t-tests were used to compare the differences between continuous variables that met the assumption of normality. Otherwise, differences between continuous variables were compared by the Mann-Whitney U test. LASSO regression was performed to identify the optimal variables for model development. The logistic regression analysis was adopted to investigate the independent predictors of in-hospital mortality of elderly cancer patients with nosocomial infections. All statistical analyses and visualizations were conducted using R software version 4.1.1 for Windows 64.0. All statistical tests were two-tailed, and statistical significance was considered at *P*<0.05.

## Results

### Demographical characteristics of the participants

**Figure 1:**
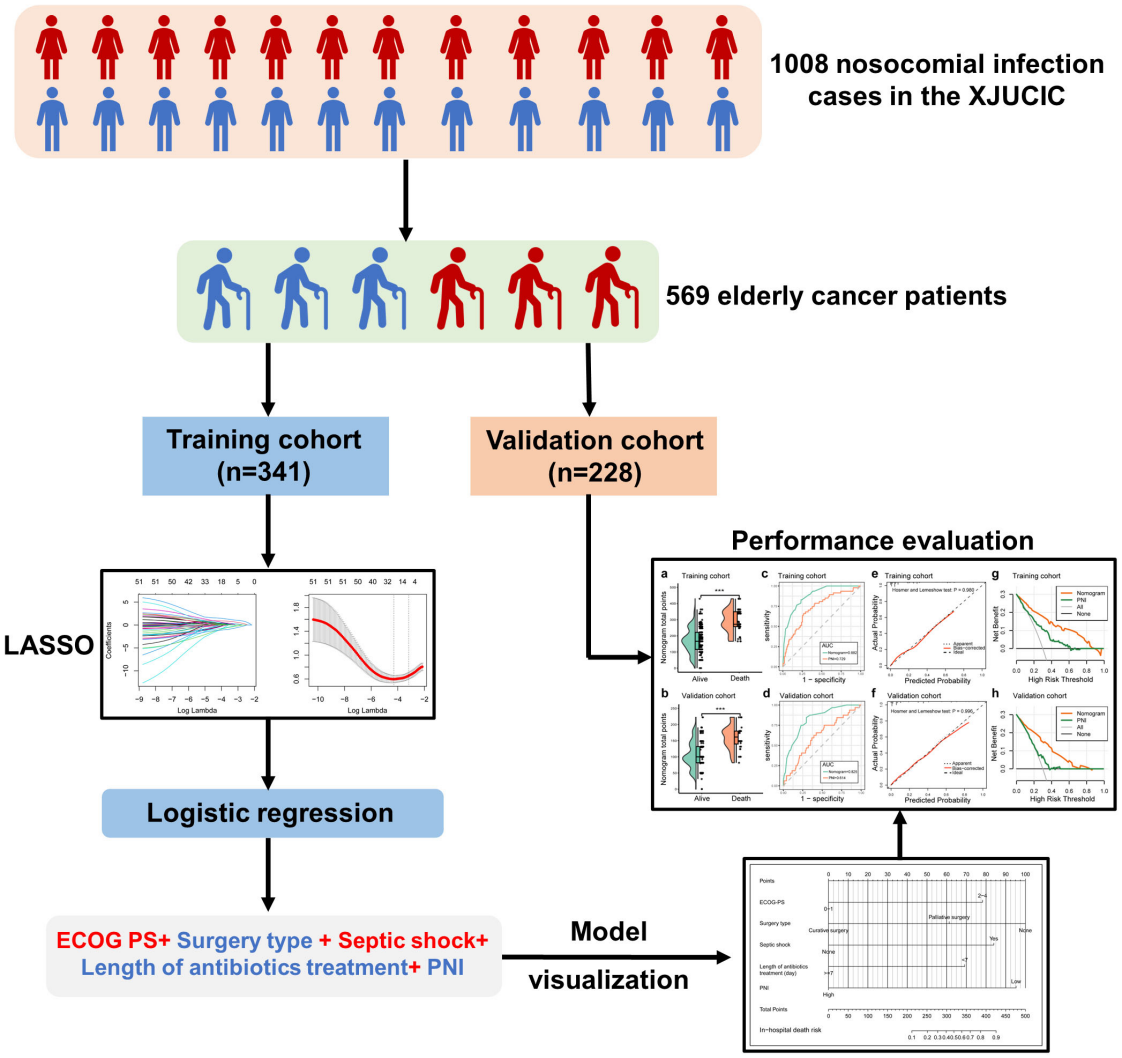
Flow chart of the study.

The study workflow is presented in **Figure 1**, with 569 elderly cancer patients enrolled. Among them, 377 (66.3%) were male and 192 (33.7%) were female, with a median age of 67 (range: 63-72) years old. Upper gastroenterology cancer was the most predominant malignancy type, accounting for 34.3% of cases, followed by lung cancer (20.7%) and gynecology cancer (10.5%). Among them, the majority of patients were diagnosed with advanced-stage disease (stage III-IV: 59.8%), but had lower ECOG-PS score (<2: 69.9%) and CCI score (<3: 52.4%). Regarding the detailed anti-neoplastic treatment, 36.2% of participants received surgery, 30.2% of patients underwent chemotherapy, and 16.5% of cases received radiotherapy, respectively. Only a small subset (7.0%) of patients was treated with concurrent chemoradiation therapy. Then, all participants were divided into three age groups and their demographical characteristics were compared. Participants older than 80 years had a higher proportion of genitourinary cancer and metastatic carcinoma (*P*<0.05; **Table S1**). Conversely, upper gastroenterology cancer was more common in patients younger than 80 years old (*P*<0.05; **Table S1**). Moreover, patients older than 80 years old had worse ECOG-PS and CCI scores, a higher proportion of cerebrovascular disease and chronic obstructive pulmonary disease (COPD) (*P*<0.05, **Table S1**). Furthermore, patients older than 80 years old received more radiotherapy and less chemotherapy than those under 80 years old (*P*<0.05, **Table S1**). **Table S1** detailed summarized the demographic features of patients in different age groups.

### The infection characteristics of nosocomial infections in elderly cancer patients

**Figure 2:**
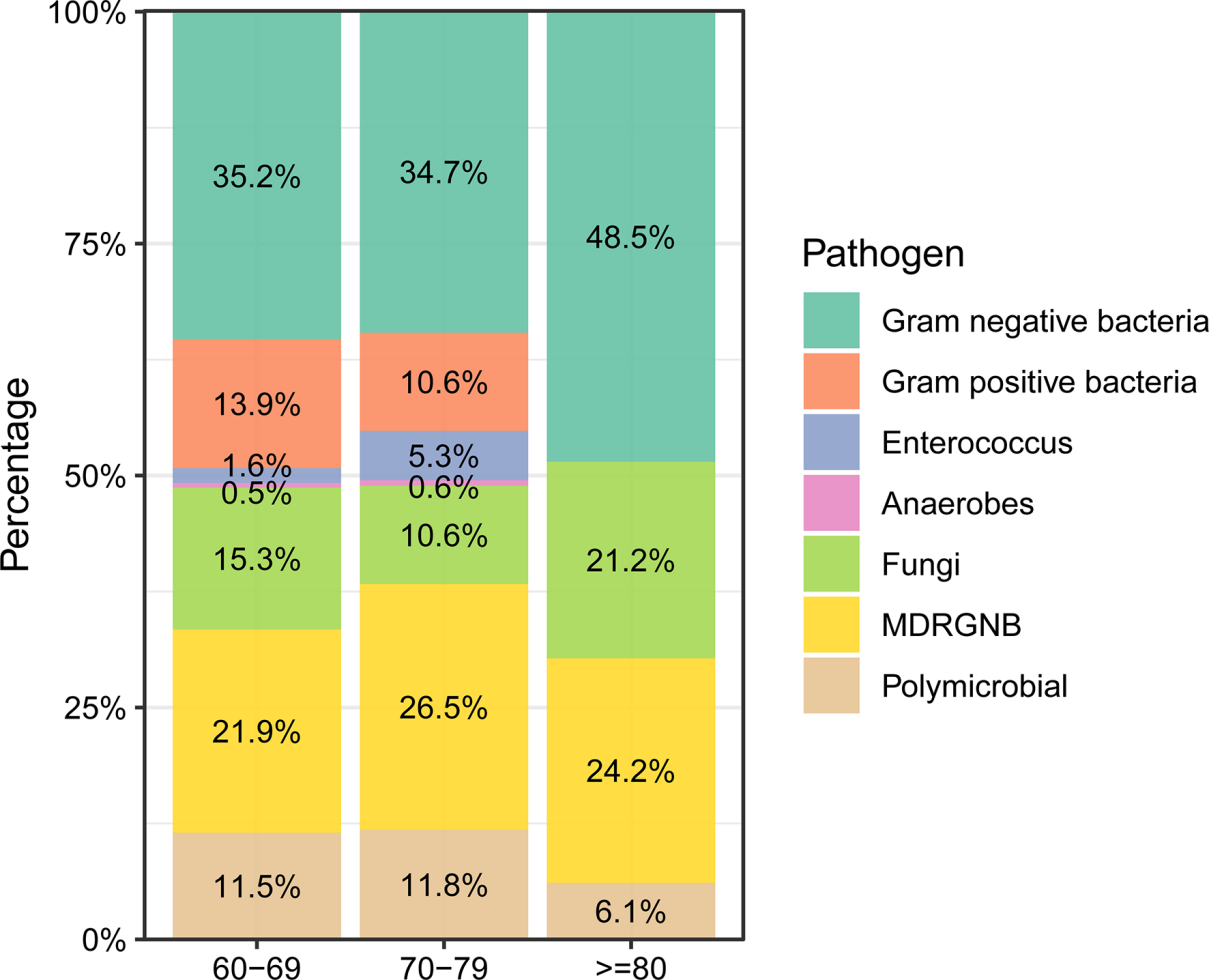
Microbiological distribution of nosocomial infections in elderly cancer patients across different age groups.

Next, we investigated the infection characteristics and microbiological distribution patterns of nosocomial infections in elderly cancer patients. Respiratory tract infection was the most frequent infection type in the participants, making up 46.6% of cases, followed by urinary tract infection (18.6%) and bloodstream infection (12.1%). Septic shock was observed in 72 patients during hospitalization. Interestingly, patients over 80 years old were found to be more susceptible to developing urinary tract infections compared to younger patients (*P*<0.05, **Table S1**). Gram-negative bacteria were the most frequently isolated pathogens in these individuals, accounting for 35.9% of participants, followed by multidrug-resistant gram-negative bacteria (MDRGNB, 23.6%) and fungi (14.2%) (**Table S2**). We also compared the microbiological distribution patterns between different age groups of cancer patients and found that gram-negative bacteria and fungi were more frequently isolated from patients over 80 years old (**Figure 2**). On the contrary, gram-positive bacteria, enterococcus, and anaerobes were only isolated from patients under 80 years old (**Figure 2**).

### The independent predictors of the in-hospital death risk of nosocomial infections in elderly cancer patients

**Figure 3:**
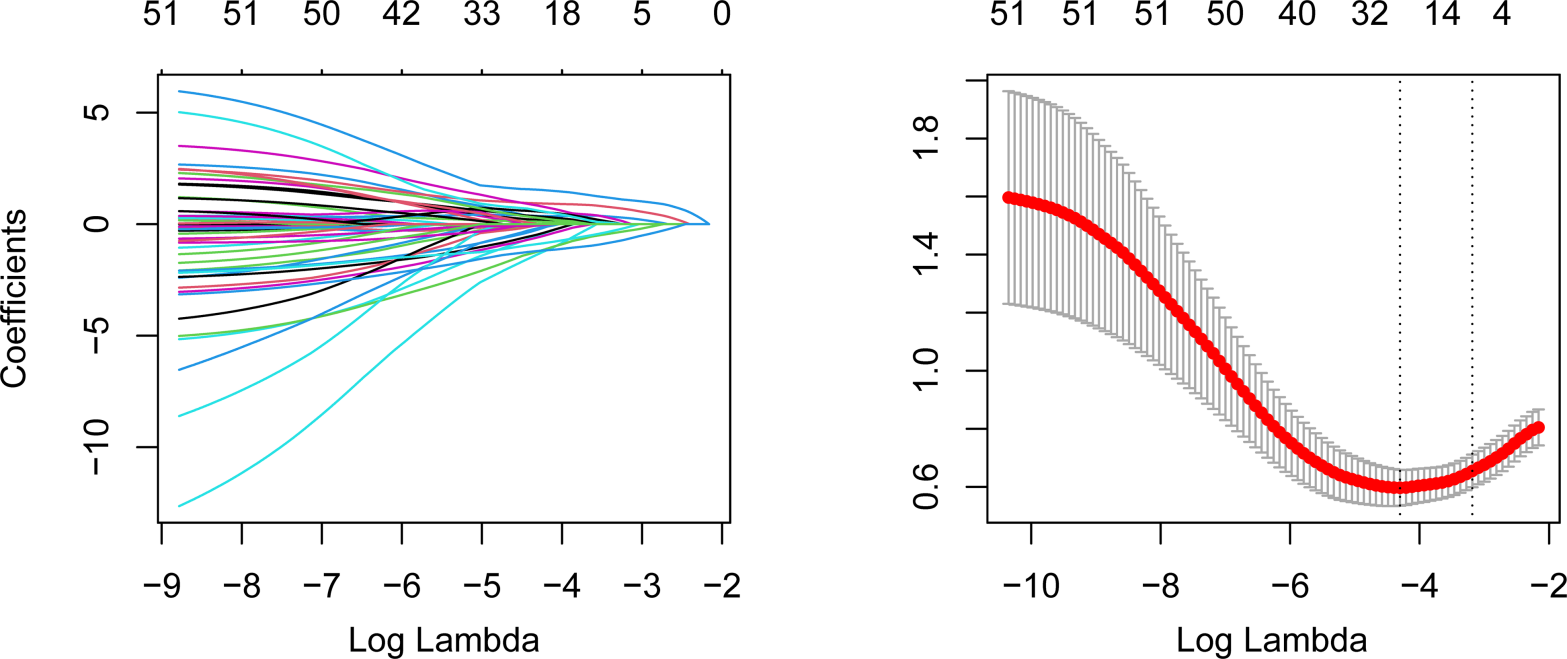
LASSO algorithm to identify the potential predictors of the in-hospital mortality of nosocomial infections in elderly cancer patients. The left panel represents the variable selection process during the LASSO penalty. The horizontal axis is the penalized parameter lambda after log transformation. The vertical axis is the coefficients of each variable. The coefficients gradually tended to zero with the increment of lambda. Eventually, variables with nonzero coefficients were selected for further analysis. The right panel is the 10-fold CV of the LASSO. LASSO, Least Absolute Shrinkage and Selection Operator; CV, cross-validation.

A total of 79 elderly cancer patients died from nosocomial infections during hospitalization, with an estimated in-hospital mortality of 13.9%. Then, we investigated the independent predictors of the in-hospital death risk of nosocomial infections in these patients. Initially, the whole cohort was subdivided into the training cohort and validation cohort, with comparable clinical parameters except for smoking status and chemotherapy (**Table 1**). The training cohort was then utilized to identify independent predictors of in-hospital death risk. Notably, several clinical variables showed significant differences between the survivors and non-survivors during nosocomial infection (**Table 1**). To overcome the issue of model overfitting in the high-dimensionality dataset, we employed the LASSO algorithm, which identified seven variables with non-zero coefficients through 10-fold CV: ECOG-PS, CCI, COPD, surgery, septic shock, length of antibiotics treatment, and PNI (**Figure 3**). Ultimately, the multivariate logistic regression analysis indicated that ECOG-PS 2-4 (odds ratio [OR]: 4.41, 95% confidence interval [CI]: 1.95-9.99; *P*<0.001) and septic shock (OR: 5.92, 95%CI: 2.43-14.44; *P*<0.001) were independent risk factors of the in-hospital death risk of nosocomial infections in the elderly patients (**Table 2**). However, we identified that underwent curative surgery (OR: 0.18, 95%CI: 0.04-0.85; *P*=0.030), length of antibiotics treatment greater than 7 days (OR: 0.21, 95%CI: 0.09-0.50; *P*<0.001), and higher PNI (OR: 0.14, 95%CI: 0.06-0.33; *P*<0.001) were independent protective factors of the in-hospital death risk of nosocomial infections in these participants (**Table 2**). CCI and COPD were significant in the univariate regression but not significant in the multivariate regression (**Table 2**).

**Table 1:** Demographical characteristics of nosocomial infections in elderly cancer patients in the different cohorts.

Characteristics	Training cohort (n=341)			Validation cohort (N=228)	*P* value^b^
	Survival(N=294)	Death(N=47)	*P* value^a^		
**Age**	68 (63-73)	67 (64-76)	0.482	67 (63-71)	0.241
60-69	199 (67.7%)	27 (57.4%)	0.365	140 (61.4%)	0.434
70-79	78 (26.5%)	17 (36.2%)		75 (32.9%)	
≥ 80	17 (5.8%)	3 (6.4%)		13 (5.7%)	
**Sex**			0.812		0.088
Female	185 (62.9%)	31 (66%)		161 (70.6%)	
Male	109 (37.1%)	16 (34%)		67 (29.4%)	
**Smoking**			0.587		**0.021**
Current/ever	160 (54.4%)	23 (48.9%)		99 (43.4%)	
Never	134 (45.6%)	24 (51.1%)		129 (56.6%)	
**Head and neck cancer**	8 (2.7%)	4 (8.5%)	0.116	9 (3.9%)	0.969
**Lung cancer**	59 (20.1%)	13 (27.7%)	0.321	46 (20.2%)	0.869
**Upper gastrointestinal cancers**	102 (34.7%)	12 (25.5%)	0.285	81 (35.5%)	0.670
**Hepatobiliary and pancreatic cancer**	11 (3.7%)	7 (14.9%)	**0.005**	14 (6.1%)	0.801
**Breast cancer**	25 (8.5%)	3 (6.4%)	0.837	15 (6.6%)	0.575
**Colorectum cancer**	27 (9.2%)	2 (4.3%)	0.399	20 (8.8%)	1.000
**Genitourinary cancer**	18 (6.1%)	1 (2.1%)	0.444	13 (5.7%)	1.000
**Gynecological cancer**	33 (11.2%)	3 (6.4%)	0.455	24 (10.5%)	1.000
**Metastasis**	5 (1.7%)	1 (2.1%)	1.000	1 (0.4%)	.311
Others^c^	6 (2%)	1 (2.1%)	1.000	5 (2.2%)	1.000
**Stage of cancer**			0.126		0.693
I-II	126 (42.9%)	14 (29.8%)		89 (39%)	
III-IV	168 (57.1%)	33 (70.2%)		139 (61%)	
**ECOG-PS**			**<0.001**		0.712
0-1	222 (75.5%)	19 (40.4%)		157 (68.9%)	
2-4	72 (24.5%)	28 (59.6%)		71 (31.1%)	
**Distant metastasis**	72 (24.5%)	23 (48.9%)	**<0.001**	69 (30.3%)	0.599
**CCI**			**<0.001**		0.852
1-2	166 (56.5%)	11 (23.4%)		121 (53.1%)	
≥ 3	128 (43.5%)	36 (76.6%)		107 (46.9%)	
**Cerebrovascular disease**	11 (3.7%)	1 (2.1%)	0.896	10 (4.4%)	0.761
**COPD**	6 (2%)	5 (10.6%)	0.008	11 (4.8%)	0.455
**T2DM**	37 (12.6%)	11 (23.4%)	0.079	27 (11.8%)	0.519
**Fever**	114 (38.8%)	27 (57.4%)	**0.024**	83 (36.4%)	0.273
**Perfusion therapy (within 30 days)**	14 (4.8%)	1 (2.1%)	0.664	9 (3.9%)	0.960
**Recent infection (within 30 days)**	13 (4.4%)	2 (4.3%)	1.000	8 (3.5%)	0.756
**FN history**	5 (1.7%)	0 (0%)	0.805	4 (1.8%)	1.000
**Chemotherapy (within 30 days)**	103 (35%)	13 (27.7%)	0.409	56 (24.6%)	**0.021**
**Radiotherapy (within 30 days)**	44 (15%)	6 (12.8%)	0.862	44 (19.3%)	0.179
**Concurrent chemoradiotherapy (within 30 days)**	23 (7.8%)	4 (8.5%)	1.000	13 (5.7%)	0.398
**Surgery type**			**<0.001**		0.953
Curative surgery	106 (36.1%)	2 (4.3%)		75 (32.9%)	
Palliative surgery	12 (4.1%)	2 (4.3%)		9 (3.9%)	
**Catheter indwelling**	165 (56.1%)	16 (34%)	**0.008**	127 (55.7%)	0.597
**Invasive procedure (within 30 days)**	187 (63.6%)	25 (53.2%)	0.228	149 (65.4%)	0.494
**Respiratory infection**	128 (43.5%)	27 (57.4%)	0.105	110 (48.2%)	0.570
**Gastrointestinal tract infection**	9 (3.1%)	1 (2.1%)	1.000	7 (3.1%)	1.000
**Urinary tract infection**	59 (20.1%)	5 (10.6%)	0.181	42 (18.4%)	1.000
**Soft tissue infection**	25 (8.5%)	0 (0%)	0.076	15 (6.6%)	0.860
**Thoracic infection**	17 (5.8%)	0 (0%)	0.183	16 (7%)	0.405
**Abdomen infection**	23 (7.8%)	2 (4.3%)	0.569	14 (6.1%)	0.703
**BSI**	33 (11.2%)	12 (25.5%)	**0.014**	24 (10.5%)	0.409
**ICU**	30 (10.2%)	6 (12.8%)	0.783	27 (11.8%)	0.732
**Mechanical ventilation**	19 (6.5%)	7 (14.9%)	0.084	18 (7.9%)	1.000
**Septic shock**	27 (9.2%)	20 (42.6%)	**<0.001**	25 (11%)	0.389
**Duration of antibiotics treatment (days)**	7 (5-11)	6 (4-11)	0.666	7 (4-10)	0.604
<7	122 (41.5%)	28 (59.6%)	**0.031**	97 (42.5%)	0.799
**Haemoglobin (g/L; normal range 115-150)**	110 (94-123)	102 (89-113)	**0.010**	107 (94-120)	0.664
< 110	156 (53.1%)	33 (70.2%)	**0.041**	116 (50.9%)	0.327
**Platelet count (×10^9^/L; normal range 125-350)**	183 (125-260)	172 (102-230)	0.275	192 (134-260)	0.312
**White-cell count (×10^9^/L; normal range 4.0-10.0)**	7.6 (5.1-10.4)	8.7 (5.7-12.0)	0.074	7.4 (5.0-10.0)	0.455
**Neutrophils count (×10^9^/L; normal range 1.8-6.3)**	6.0 (3.6-8.5)	7.9 (4.2-10.8)	0.042	5.8 (3.4-8.6)	0.295
**Lymphocytes count (×10^9^/L; normal range 1.1-3.2)**	0.9 (0.6-1.2)	0.7 (0.5-1.0)	0.061	0.9 (0.5-1.3)	0.841
< 1.0	166 (56.5%)	34 (72.3%)	0.058	134 (58.8%)	1.000
**Monocyte (×10^9^/L; normal range 0.1-0.6)**	0.4 (0.2-0.6)	0.4 (0.2-0.6)	0.818	0.4 (0.3-0.6)	0.752
**PCT (ng/mL; normal range 0-0.5)**	0.4 (0.4-0.5)	0.7 (0.4-4.6)	**<0.001**	0.4 (0.4-0.8)	0.749
≥ 1.0	57 (19.4%)	21 (44.7%)	**<0.001**	46 (20.2%)	0.509
**Albumin (g/L; normal range 40-55)**	33 (29-37)	30 (28-33)	**<0.001**	33 (29-38)	0.396
< 30.0	82 (27.9%)	27 (57.4%)	**<0.001**	69 (30.3%)	0.736
**PNI**	37.7 (32.8-43.4)	34.0 (30.8-36.9)	**<0.001**	38.4 (33.2-43.1)	0.297
Low	121 (41.2%)	37 (78.7%)	**<0.001**	100 (43.9%)	0.620

ECOG-PS, Eastern Cooperative Oncology Group performance status; CCI, Charlson Co-morbidity Index score; COPD, chronic obstructive pulmonary disease; T2DM, type 2 diabetes mellitus; FN, febrile neutropenia; BSI, bloodstream infection; ICU, intensive care unit; CRP, C-reactive protein; PCT, procalcitonin; PNI, prognostic nutritional index.

a P value for univariate analysis of the training cohort

b P value for clinical characteristics analysis between the training cohort and validation cohort

c Others: primitive neuroectodermal tumor (4 patients), thymic carcinoma and duodenal carcinoma two patients each, malignant teratoma, melanoma, adrenal carcinoma, and carcinoid cancer of appendix one patient each.

Bolded values indicate statistical significance.

**Table 2:** The results of the univariate and multivariate logistic regression analyses in the training cohort.

Characteristics	Univariate analysis		Multivariate analysis	
	OR (95%CI)	*P* value	OR (95%CI)	*P* value
ECOG-PS				
0-1	Ref		Ref	
2-4	**4.54 (2.40-8.62)**	**<0.001**	**4.41 (1.95-9.99)**	**<0.001**
CCI				
1-2	Ref		Ref	
≥3	**4.24 (2.08-8.66)**	**<0.001**	2.19 (0.92-5.18)	0.076
COPD				
None	Ref		Ref	
Yes	**5.71 (1.67-19.55)**	**0.005**	4.97 (0.88-27.94)	0.069
Surgery type				
	Ref		Ref	
Curative surgery	**0.08 (0.02-0.33)**	**<0.001**	**0.18 (0.04-0.85)**	**0.030**
Palliative surgery	0.68 (0.15-3.16)	0.625	0.43 (0.06-2.90)	0.386
Septic shock				
None	Ref		Ref	
Yes	**7.33 (3.63-14.76)**	**<0.001**	**5.92 (2.43-14.44)**	**<0.001**
Duration of antibiotics treatment (days)				
<7	Ref		Ref	
>=7	**0.48 (0.26-0.90)**	**0.022**	**0.21 (0.09-0.50)**	**<0.001**
PNI				
Low	Ref		Ref	
High	**0.19 (0.09-0.39)**	**<0.001**	**0.14 (0.06-0.33)**	**<0.001**

OR, odds ratio; CI, confidence interval; ECOG-PS, Eastern Cooperative Oncology Group performance status; CCI, Charlson Co-morbidity Index score; COPD, chronic obstructive pulmonary disease; PNI, prognostic nutritional index.

Bolded values indicate statistical significance.

### The development and assessment of a nomogram to predict the in-hospital death risk of nosocomial infections in elderly cancer patients

**Figure 4:**
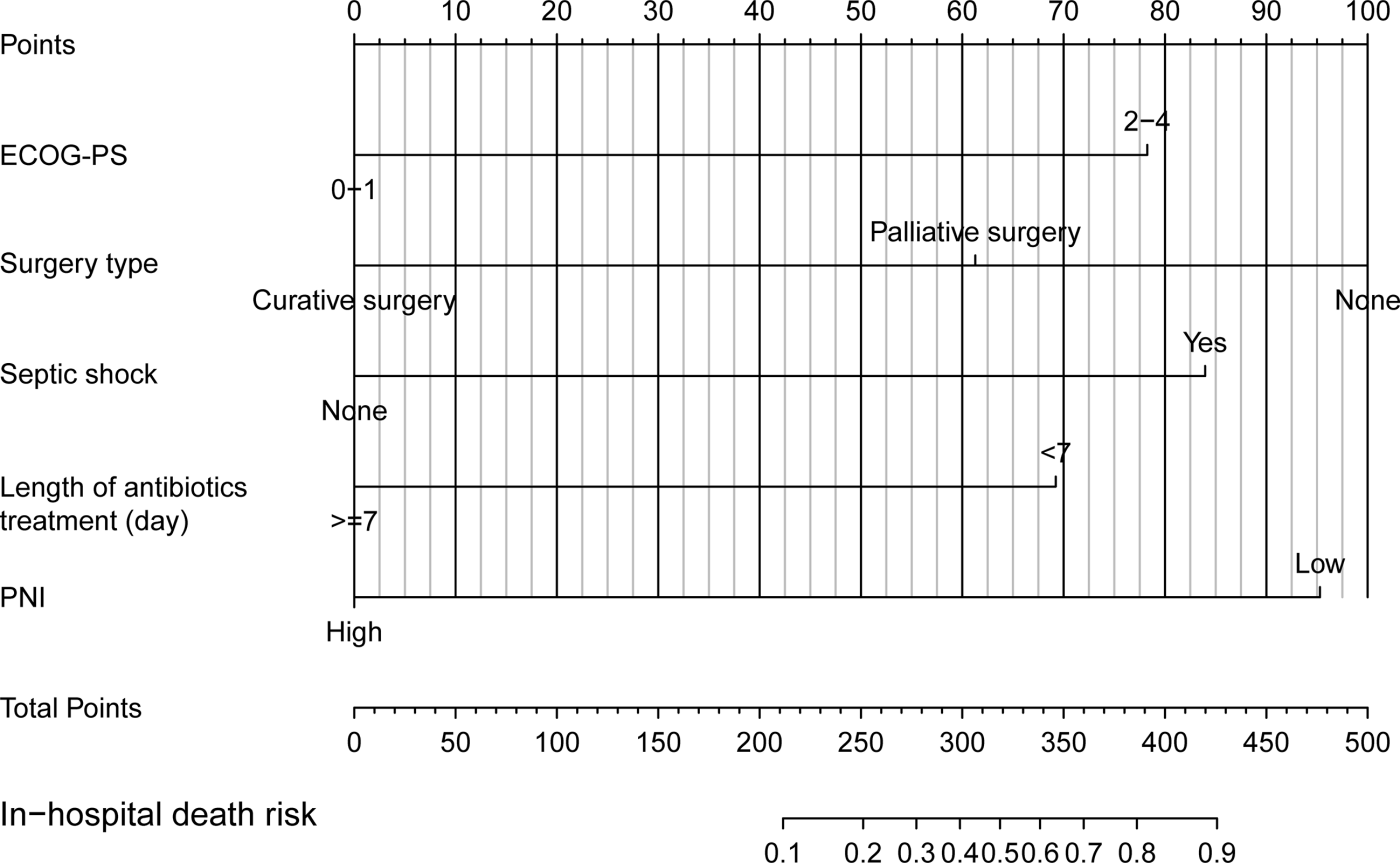
Nomogram for predicting in-hospital mortality of nosocomial infections in elderly cancer patients.

**Figure 5:**
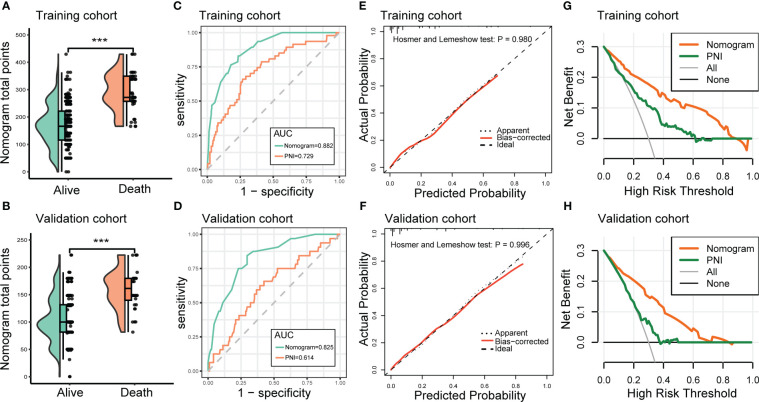
Evaluation of the performance of the nomogram in predicting the in-hospital mortality of nosocomial infections in elderly cancer patients. **(A, B)** Raincloud plots to show the total points derived from the nomogram of patients with different outcomes in the training and validation cohorts. **(C, D)** ROC curves to depict the discrimination ability of the nomogram in the training and validation cohorts. **(E, F)** Calibration curves to assess the calibration ability of the nomogram in the training and validation cohorts. **(G, H)** DCA to demonstrate the net clinical benefits of the nomogram in the training and validation cohorts. ROC, receiver operating characteristics; DCA, decision curve analysis.

Next, we developed a nomogram to predict the in-hospital death risk of nosocomial infections in elderly cancer patients based on the above five independent predictors. As vividly illustrated in **Figure 4**, we could estimate the total points of each participant based on the above variables in the nomogram, thus calculating their corresponding in-hospital death risk. Therefore, clinicians can identify patients at high risk of in-hospital death in a timely manner according to the nomogram. Besides, we observed that patients who died during the hospitalization after nosocomial infections were correlated to higher nomogram total points both in the training and validation cohorts (**Figures 5A, B**). ROC curves also yield excellent AUC values of the nomogram in the training (AUC=0.882) and validation (AUC=0.825) cohorts, suggesting its satisfied discrimination ability (**Figure 5C, D**; **Table 3**). Furthermore, the calibration ability of the nomogram was also evaluated by calibration curves, which showed higher consistencies between the predicted and actual in-hospital death risk in both cohorts (**Figures 5E, F**). The results of the Hosmer-Lemeshow test further supported the good calibration ability of the nomogram (*P*>0.05 both in the training and validation cohorts). Ultimately, DCA was utilized to assess the net clinical benefits of the nomogram when it was applicated in clinical practice (**Figures 5G, H**). The results indicated that the nomogram will yield significant clinical net benefits to participants within certain risk thresholds. Taken together, the constructed nomogram is a well and easy tool to predict the in-hospital death risk of nosocomial infections in elderly cancer patients.

**Table 3:** The performance of the novel risk classifier in predicting the in-hospital mortality of nosocomial infections in elderly cancer patients.

	AUC (95% CI)	*P* value ^a^	Sensitivity	Specificity
Training cohort				
PNI	0.729 (0.650-0.808)	–	0.731	0.660
Nomogram	0.882 (0.837-0.927)	**<0.001**	0.765	0.830
Validation cohort				
PNI	0.614 (0.508-0.720)	–	0.577	0.656
Nomogram	0.825 (0.753-0.897)	**<0.001**	0.704	0.844

AUC, area under the curve; CI, confidence interval; PNI, prognostic nutritional index.

a P values were calculated by using the Bootstrap method in the “pROC” package.

Bolded values indicate statistical significance.

## Discussion

In the current study, we investigated the clinical characteristics, microbiological distribution, and prognostic factors of nosocomial infections in elderly cancer patients. Most importantly, we developed a nomogram to predict the in-hospital death risk of these patients, with excellent performance being observed both in the training and validation cohorts. Consistent with our previous work and other researchers’ work, gram-negative bacteria remain the most predominant causative pathogens of nosocomial infections in elderly cancer patients (Huang et al., 2011; Gudiol et al., 2016; Antonio et al., 2019; Jiang et al., 2022). Besides, the MDR phenomenon is also common in these populations, accounting for 24.0% of infection episodes, which is higher than the 12.7% reported in a previous publication (Antonio et al., 2019). Regarding the detailed infection type, we identified that patients aged over 80 years were more prone to develop urinary tract infections compared to younger patients, which is good in line with the study conducted by Antonio and colleagues (Antonio et al., 2019). On the one hand, the majority of the elderly patients in this study had poor ECOG-PS scores, indicating that their physical activity is severely limited. On the other hand, we identified that a higher proportion of older cancer patients suffered from genitourinary cancer, which will potentially increase the risk of urinary tract infection after anti-cancer treatment. Urinary tract infection is one of the most common complications after radical cystectomy and orthotopic neobladder reconstruction (Kim et al., 2016). In a retrospective study, Kim and colleagues reported that febrile urinary tract infections occurred in 17.6% of bladder cancer patients after radical cystectomy and ileal neobladder reconstruction (Kim et al., 2016).

In this study, it was found that 13.9% of elderly cancer patients died due to nosocomial infections during hospitalization, which is lower than previously reported (Aydemir et al., 2013; Antonio et al., 2019). We attribute this to the fact that only 1.6% of the participants in our study experienced neutropenia, which is often associated with a poor clinical outcome of infections in patients with malignancy (Jiang et al., 2020a). In contrast, in Aydemir’s study, all participants experienced neutropenia, and 25.9% of patients had prolonged neutropenia (Aydemir et al., 2013). Furthermore, even in Antonio’s study, the proportion of neutropenia cases (14.6%) was significantly higher than in our cohort (Antonio et al., 2019). We found that elderly cancer patients with poor ECOG-PS or who experienced septic shock are correlated with higher in-hospital mortality. On the contrary, underwent curative surgery, length of antibiotics treatment greater than 7 days, and higher PNI were associated with lower case fatality in these participants. The significance of septic shock in the prognosis of nosocomial infections in cancer patients is well documented (Chen et al., 2017; Palacios-Baena et al., 2017; Antonio et al., 2019; Jiang et al., 2020a; Gudiol et al., 2021; Jiang et al., 2022). Septic shock is defined as a life-threatening subset of sepsis that can cause multiorgan failure and is associated with higher mortality (Singer et al., 2016; Cuenca et al., 2022). Sepsis and septic shock are among the most common reasons for ICU admission in patients with malignancy (Cuenca et al., 2022). It is reported that cancer patients who experienced septic shock have a 1.85-fold hospital mortality compared to the general population (Buchman et al., 2020a; Buchman et al., 2020b; Awad et al., 2021; Manjappachar et al., 2022). Cancer patients who underwent curative surgery were at the early stage of the disease and generally correlated with well physical activity and respiratory and circulation function. Besides, a previous study has shown that intra-abdominal infection after curative surgery did not damage the long-term survival benefits of cancer patients (Tu et al., 2019). However, considering the limited sample size in this subset, the prospective study should be designed to provide reliable evidence in this field. PNI is derived from the serum albumin concentration and lymphocyte count, which is frequently adopted to reflect the nutritional and immunological status of patients (Xiao et al., 2022). A growing number of studies have elucidated that PNI is significantly correlated with the prognosis of patients with malignancies(Wang et al., 2018; Jiang et al., 2020c; Karimi et al., 2021). Emerging evidence also revealed that PNI is also significantly correlated with the prognosis of infectious disease(Doganci et al., 2020; Karimi et al., 2021; Xiao et al., 2022). In the current study, we identified that PNI was independently correlated with the in-hospital mortality of nosocomial infections in elderly cancer patients. Hence, PNI could serve as a reliable nutritional and immunological index to guide the management of nosocomial infections in these patients.

Although we identified several clinical factors that were associated with the in-hospital death risk of nosocomial infections in elderly cancer patients, the predictive ability of a single marker was inferior to the combination index. Therefore, we developed a novel risk classifier based on these variables to predict the in-hospital death risk of nosocomial infections in elderly cancer patients. As expected, the constructed nomogram yielded excellent discrimination ability and calibration ability in both the training and validation cohorts. More importantly, we applied DCA to evaluate the net clinical benefits of the nomogram in guiding clinical decisions. Strikingly, the nomogram showed superior net clinical benefits compared to PNI. These results demonstrate that we have developed a reliable risk classifier that can accurately predict the in-hospital death risk of nosocomial infections in elderly cancer patients.

Despite the advantages of our study, several inevitable shortcomings also exist in the current study. Firstly, due to the retrospective design of our study, selection and informative biases could not be completely eliminated. Secondly, although many factors, particularly inflammatory parameters, are known to affect the prognosis of nosocomial infection in cancer patients, the lack of clear medical chart recordings for these parameters, such as C-reactive protein (CRP) and serum cytokines, hindered their inclusion in our study. Therefore, future prospective studies are required to investigate the correlation between these parameters and the prognosis of nosocomial infections in elderly cancer patients. Finally, even though our nomogram demonstrated excellent performance, further external validation cohorts are necessary to assess its generalizability.

## Conclusions

In summary, nosocomial infections are prevalent in elderly cancer patients and are associated with higher in-hospital mortality. Gram-negative bacteria remain the most predominant causative pathogens, and the MDR phenomenon is not rare. Besides, different age groups are correlated with distinct infection types. Most importantly, we developed a novel and reliable risk classifier that could accurately predict the in-hospital mortality of nosocomial infections in these individuals based on ECOG-PS, surgery type, the existence of septic shock, length of antibiotics treatment, and PNI.

## Data availability statement

The original contributions presented in the study are included in the article/**Supplementary Material**. Further inquiries can be directed to the corresponding authors.

## Ethics statement

The studies involving human participants were reviewed and approved by The ethics committee of the Frist Affiliated Hospital of Xi’an Jiaotong University. The ethics committee waived the requirement of written informed consent for participation.

## Author contributions

YY, TT, and AJ conceived the study. AJ, YL, NZ, and XS participated in the literature search, study design, data collection, data analysis, and wrote the manuscript. NL, JW, and HG participated in data collection and analysis. ZR and XL proposed the study and participated in its design. XF participated in the study design and helped with data analysis. All authors contributed to the article and approved the submitted version.
