# Supplementary material for: A novel risk classifier to predict the in-hospital death risk of nosocomial infections in elderly cancer patients
Source: Front Cell Infect Microbiol. 2023 May 10;13:1179958. doi: 10.3389/fcimb.2023.1179958 (PMC10206213; doi:10.3389/fcimb.2023.1179958)
Supplement: Supplementary file 1 [file DataSheet_1.pdf]

Table S1. Demographical and clinical characteristics in geriatric cancer patients according to age.

Characteristics	Whole cohorts (N=569)	Age, years			*P* value
		60-69 (N=366)	70-79 (N=170)	≥80 (N=33)	
**Sex**					0.756
Female	377 (66.3%)	245 (66.9%)	112 (65.9%)	20 (60.6%)	
Male	192 (33.7%)	121 (33.1%)	58 (34.1%)	13 (39.4%)	
**Tobacco use**					0.838
Current/ever	282 (49.6%)	178 (48.6%)	87 (51.2%)	17 (51.5%)	
Never	287 (50.4%)	188 (51.4%)	83 (48.8%)	16 (48.5%)	
**Head and neck cancer**	21 (3.7%)	15 (4.1%)	5 (2.9%)	1 (3%)	0.787
**Lung cancer**	118 (20.7%)	75 (20.5%)	35 (20.6%)	8 (24.2%)	0.877
**Upper gastrointestinal cancers**	195 (34.3%)	130 (35.5%)	61 (35.9%)	4 (12.1%)	**0.022**
**Hepatobiliary and pancreatic cancer**	32 (5.6%)	20 (5.5%)	10 (5.9%)	2 (6.1%)	0.975
**Breast cancer**	43 (7.6%)	31 (8.5%)	10 (5.9%)	2 (6.1%)	0.542
**Colorectum cancer**	49 (8.6%)	30 (8.2%)	17 (10%)	2 (6.1%)	0.681
**Genitourinary cancer**	32 (5.6%)	11 (3%)	14 (8.2%)	7 (21.2%)	**<0.001**
**Gynecological cancer**	60 (10.5%)	42 (11.5%)	14 (8.2%)	4 (12.1%)	0.501
**Metastasis**	7 (1.2%)	4 (1.1%)	1 (0.6%)	2 (6.1%)	**0.031**
**Others**[a]	12 (2.1%)	8 (2.2%)	3 (1.8%)	1 (3%)	0.885
**Stage of cancer**					0.468
I-II	229 (40.2%)	141 (38.5%)	75 (44.1%)	13 (39.4%)	
III-IV	340 (59.8%)	225 (61.5%)	95 (55.9%)	20 (60.6%)	
**ECOG-PS**					**<0.001**
0-1	398 (69.9%)	278 (76%)	107 (62.9%)	13 (39.4%)	
2-4	171 (30.1%)	88 (24%)	63 (37.1%)	20 (60.6%)	
**Distant metastasis**	164 (28.8%)	107 (29.2%)	48 (28.2%)	9 (27.3%)	0.952
**CCI**					0.094
1-2	298 (52.4%)	201 (54.9%)	85 (50%)	12 (36.4%)	
>=3	271 (47.6%)	165 (45.1%)	85 (50%)	21 (63.6%)	
**Cerebrovascular disease**	22 (3.9%)	15 (4.1%)	3 (1.8%)	4 (12.1%)	**0.017**
**COPD**	22 (3.9%)	7 (1.9%)	10 (5.9%)	5 (15.2%)	**<0.001**
**T2DM**	75 (13.2%)	46 (12.6%)	22 (12.9%)	7 (21.2%)	0.370

Table S1. Demographical and clinical characteristics in geriatric cancer patients according to age.

Characteristics	Whole cohorts (N=569)	Age, years			*P* value
		60-69 (N=366)	70-79 (N=170)	≥80 (N=33)	
**Fever**	224 (39.4%)	145 (39.6%)	71 (41.8%)	8 (24.2%)	0.167
**Perfusion therapy (within 30 days)**	24 (4.2%)	15 (4.1%)	9 (5.3%)	0 (0%)	0.377
**Recent infection (with 30 days)**	23 (4.0%)	14 (3.8%)	8 (4.7%)	1 (3%)	0.850
**FN history**	9 (1.6%)	4 (1.1%)	5 (2.9%)	0 (0%)	0.211
**Chemotherapy (within 30 days)**	172 (30.2%)	134 (36.6%)	37 (21.8%)	1 (3%)	**<0.001**
**Radiotherapy (within 30 days)**	94 (16.5%)	47 (12.8%)	35 (20.6%)	12 (36.4%)	**<0.001**
**Concurrent chemoradiotherapy (within 30 days)**	40 (7.0%)	32 (8.7%)	8 (4.7%)	0 (0%)	0.063
**Surgery type**					0.105
Curative surgery	183 (32.2%)	126 (34.4%)	52 (30.6%)	5 (15.2%)	
Palliative surgery	23 (4.0%)	11 (3%)	10 (5.9%)	2 (6.1%)	
**Catheter indwelling**	308 (54.1%)	205 (56%)	93 (54.7%)	10 (30.3%)	**0.018**
**Invasive procedure (with 30 days)**	361 (63.4%)	235 (64.2%)	107 (62.9%)	19 (57.6%)	0.741
**Respiratory infection**	265 (46.6%)	163 (44.5%)	84 (49.4%)	18 (54.5%)	0.367
**Gastrointestinal tract infection**	17 (3.0%)	14 (3.8%)	3 (1.8%)	0 (0%)	0.249
**Urinary tract infection**	106 (18.6%)	56 (15.3%)	39 (22.9%)	11 (33.3%)	**0.009**
**Soft tissue infection**	40 (7.0%)	28 (7.7%)	12 (7.1%)	0 (0%)	0.258
**Thoracic infection**	33 (5.8%)	25 (6.8%)	8 (4.7%)	0 (0%)	0.211
**Abdomen infection**	39 (6.9%)	32 (8.7%)	7 (4.1%)	0 (0%)	**0.039**
**BSI**	69 (12.1%)	48 (13.1%)	17 (10%)	4 (12.1%)	0.590
**ICU**	63 (11.1%)	39 (10.7%)	19 (11.2%)	5 (15.2%)	0.732
**Mechanical ventilation**	44 (7.7%)	24 (6.6%)	17 (10%)	3 (9.1%)	0.364
**Septic shock**	72 (12.7%)	48 (13.1%)	20 (11.8%)	4 (12.1%)	0.905
**Duration of antibiotics treatment (days)**	7 (4-11)	7 (4-11)	7 (5-11)	7 (5-12)	0.893
<7	247 (43.4%)	163 (44.5%)	69 (40.6%)	15 (45.5%)	0.672
**Haemoglobin (g/L; normal range 115-150)**	107 (94-121)	108 (93-122)	107 (93-120)	110 (94-128)	0.545
< 110	305 (53.6%)	195 (53.3%)	94 (55.3%)	16 (48.5%)	0.756
**Platelet count (×10^9/L; normal range 125-350)**	184 (126-259)	186 (126-258)	179 (125-264)	189 (150-250)	0.830

Table S1. Demographical and clinical characteristics in geriatric cancer patients according to age.

Characteristics	Whole cohorts (N=569)	Age, years			*P* value
		60-69 (N=366)	70-79 (N=170)	≥80 (N=33)	
**White-cell count (×10^9/L; normal range 4.0-10.0)**	7.6 (5.1-10.5)	7.5 (4.9-10.5)	7.8 (5.4-11.1)	7.0 (5.6-10.0)	0.692
**Neutrophils count (×10^9/L; normal range 1.8-6.3)**	5.9 (3.5-8.8)	5.9 (3.4-8.6)	6.6 (3.9-9.3)	5.6 (4.2-8.3)	0.667
**Lymphocytes count (×10^9/L; normal range 1.1-3.2)**	0.9 (0.5-1.3)	0.9 (0.6-1.2)	0.9 (0.5-1.3)	0.8 (0.5-1.4)	0.706
< 1.0	334 (58.7%)	212 (57.9%)	103 (60.6%)	19 (57.6%)	0.836
**Monocyte (×10^9/L; normal range 0.1-0.6)**	0.4 (0.2-0.6)	0.4 (0.2-0.6)	0.4 (0.3-0.6)	0.4 (0.3-0.6)	0.928
**PCT (ng/mL; normal range 0-0.5)**	0.4 (0.4-0.73)	0.4 (0.4-0.7)	0.4 (0.4-1.0)	0.4 (0.4-1.6)	0.216
≥ 1.0	124 (21.8%)	76 (20.8%)	41 (24.1%)	7 (21.2%)	0.680
**Albumin (g/L; normal range 40-55)**	33 (29-37)	33 (29-38)	32 (29-37)	33 (30-38)	0.180
< 30.0	178 (31.3%)	113 (30.9%)	59 (34.7%)	6 (18.2%)	0.166
**PNI**	37.6 (32.7-43.0)	38.0 (32.6-43.1)	36.3 (32.2-42.3)	37.4 (34.6-43.4)	0.245
Low	258 (45.3%)	152 (41.5%)	90 (52.9%)	16 (48.5%)	**0.044**

Abbreviations: ECOG-PS, Eastern Cooperative Oncology Group performance status; CCI, Charlson Co-morbidity Index score; COPD, chronic obstructive pulmonary disease; T2DM, type 2 diabetes mellitus; FN, febrile neutropenia; BSI, bloodstream infection; ICU, intensive care unit; CRP, C-reactive protein; PCT, procalcitonin; PNI, prognostic nutritional index.

[a] Others: primitive neuroectodermal tumor (4 patients), thymic carcinoma and duodenal carcinoma two patients each, malignant teratoma, melanoma, adrenal carcinoma, and carcinoid cancer of appendix one patient each.

Bolded values indicate statistical significance.

Table S2. Causative agents of all nosocomial infection episodes in geriatric cancer patients.

**Causative organisms**	**N (%)**
**Gram-negative bacteria**	204 (35.9)
Escherichia coli	53 (9.3)
Klebsiella pneumoniae	44 (7.7)
Pseudomonas aeruginosa	47 (8.3)
Enterobacter spp.	20 (3.5)
Klebsiella oxytoca	3 (0.5)
Proteus mirabilis	6 (1.1)
Haemophilus spp	26 (4.6)
Others	5 (0.9)
**Gram-positive bacteria**	69 (12.1)
Staphylococcus aureus	32 (5.6)
Methicillin-resistant *S. aureus*	2 (0.4)
Streptococcus pneumoniae	19 (3.3)
Coagulase-negative *staphylococci*	7 (1.2)
Streptococcus anginosus	5 (0.9)
Others	4 (0.7)
**Enterococcus**	15 (2.6)
E. faecalis	7 (1.2)
E. faecium	4 (0.7)
Enterococcus spp	4 (0.7)
**Anaerobes**	3 (0.5)
**Fungi**	81 (14.2)
Candida albicans	55 (9.7)
Candida spp.	7 (1.2)
Aspergillus flavus	4 (0.7)
Eurotium	15 (2.6)
**MDRGNB**	134 (23.6)
ESBL-producing Enterobacteriaceae	93 (16.3)
MDR *Pseudomonas aeruginosa*	11 (1.9)
Acinetobacter baumannii	16 (2.8)
Stenotrophomonas maltophilia	9 (1.6)
Carbapenem-resistant Enterobacteriaceae	3 (0.5)
**Polymicrobial**	64 (11.2)

Abbreviations: MDRGNB，multidrug-resistant gram-negative bacilli；ESBL, extended-spectrum β-lactamase; MDR: multidrug-resistant.

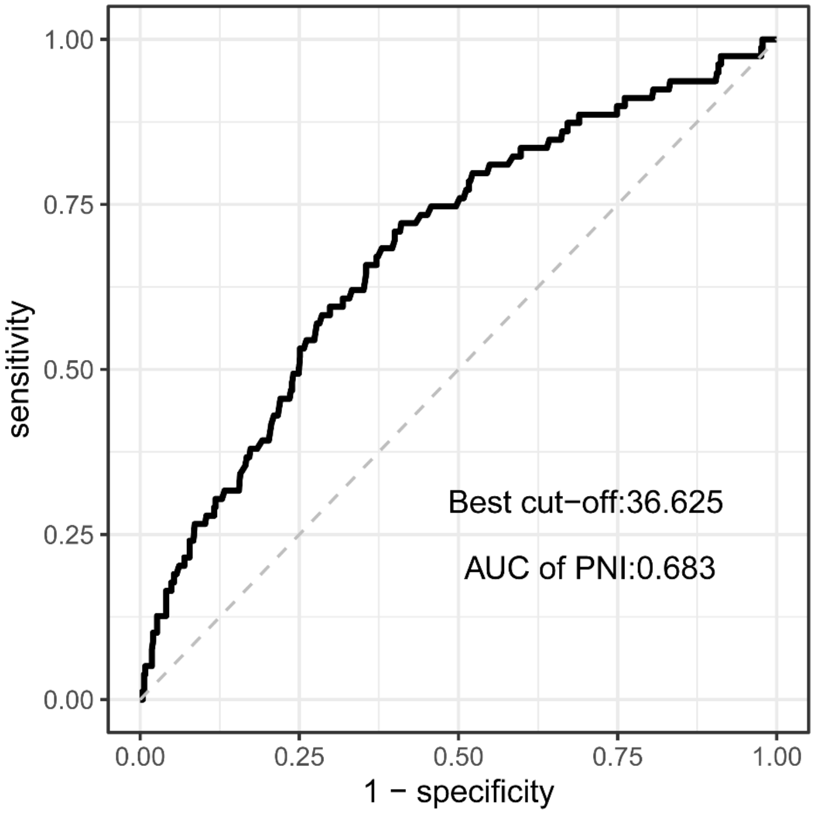

Figure S1. ROC curve to determine the best cut-off value of PNI. ROC, receiver operating characteristics; PNI, prognostic nutritional index.
